# Spatio-temporal distribution and aggregation analysis of road traffic fatalities in Shandong Province, China, 2012–2022

**DOI:** 10.3389/fpubh.2025.1711959

**Published:** 2025-12-15

**Authors:** Tao Wang, Zi-Long Lu, Jie Chu, Qi Li, Zhi-ying Yao, Xiao-Lei Guo, Cun-Xian Jia

**Affiliations:** 1 Phase 1 Clinical Trial Center, Deyang People’s Hospital, Deyang, Sichuan, China; 2 Department of Epidemiology, School of Public Health, Cheeloo College of Medicine, Shandong University, Jinan, China; 3 Institute for Chronic Non-communicable Diseases Control and Prevention, Shandong Center for Disease Control and Prevention, Jinan, Shandong, China

**Keywords:** road traffic fatalities, Shandong province, temporal distribution, spatial autocorrelation, spatio-temporal aggregation

## Abstract

**Objective:**

This study aimed to analyze the temporal and spatial distribution, as well as spatio-temporal aggregation, of road traffic fatalities in Shandong Province, China, from 2012 to 2022, with the aim of establishing scientific foundation for crafting customized intervention strategies and preventive actions to mitigate road traffic fatalities.

**Methods:**

Data were obtained from the Chinese Center for Disease Control and Prevention Population Death Information Registration Management System. Statistical analyses, including composition ratios, chi-square tests, spatial autocorrelation analyses, and spatio-temporal aggregation, were conducted. Software tools, such as Excel, Geoda, and SaTScan v10.1.2, were utilized for data analysis.

**Results:**

The study showed pedestrians were the most affected group (55.18%), followed by motorized drivers, non-motorized drivers, and passengers. The temporal distribution showed cyclical trends, with the largest number of deaths in autumn. Passengers had a higher number of deaths during leave in lieu (*χ^2^* = 12.247, *p* = 0.007) and vacation (*χ^2^* = 17.599, *p* = 0.001) than other subgroups. The spatial distribution identified varying hotspots and cold spots across different cities in Shandong Province. The spatial autocorrelation analysis indicated unique patterns for different groups of road traffic fatalities. Spatio-temporal cluster analysis indicated that a notable and novel finding was the emergence of non-motorized drivers as the newest spatio-temporal agglomeration in southwestern Shandong, while that of motorized drivers was distinctly located in the Jiaodong Peninsula.

**Conclusion:**

Targeted measures in high-risk areas and peak periods have reduced road traffic fatalities. Legislative efforts and educational campaigns have improved road safety; however, challenges with e-bikes require focused interventions.

## 1 Introduction

Road traffic injuries (RTIs) are a global public health problem, with fatalities being the most serious consequences of RTIs. The 2018 Global Status Report on Road Safety (1) published by the World Health Organization (WHO) reveals that RTIs are the eighth leading cause of death for the general population worldwide and the leading cause of death for people aged 5–29 years. Although low- and middle-income countries account for only 60% of all motor vehicles worldwide, they account for 93% of global road traffic fatalities (2). In China, RTIs are the most common cause of injury-related deaths, with a mortality rate of 14.23/100,000 (3). Road traffic crashes can result in huge economic losses and casualties. In 2021, the WHO reported that economic costs of road traffic crashes accounted for 3% of the gross domestic product (GDP) of most countries. This has also caused enormous economic losses to China. In 2017, the total economic cost of road traffic accidents in China was 490.1 billion RMB, accounting for 0.60% of the total GDP of that year (4). RTIs are estimated to cost the global economy $1.8 trillion from 2015 to 2030 (5). RTIs seriously threaten people’s lives and property and impose a heavy burden on society, families, and individuals.

Due to the serious consequences of RTIs, both the United Nations and WHO have issued guidelines, resulting in a significant decrease in the incidence and mortality rates of road traffic crashes in some countries (6–8). In contrast, in low- and middle-income countries, the incidence and mortality rates of road traffic crashes are still increasing (9). Since 2004, China has introduced relevant laws and regulations to improve road traffic safety, including the Road Traffic Safety Law of the People’s Republic of China (2004), strict penalties for drunk driving (2009), and the criminalization of drunk driving (2011). The mortality rate from road traffic crashes has declined in China, but the burden of disease caused by them remains significant (10, 11). In China, RTIs mainly occur in economically developed provinces such as Zhejiang and Jiangsu (12), which are highly prone to road traffic crashes due to high motor vehicle ownership and large traffic flow. Currently, China’s economy is experiencing rapid development, with road construction and the number of motor vehicles growing quickly. Although vehicles provide users with a convenient way to travel, they pose risks to traffic safety. In addition to traditional modes of transportation, new ones such as e-bikes, shared bicycles, and shared cars have become widely used in China, and RTIs involving them are increasing yearly (13, 14). A study in Zhejiang Province found that road traffic crashes caused by e-bikes accounted for one-third of all traffic crashes from 2008 to 2011 (15). Therefore, the increase in the number of vehicles and the emergence of new modes of transportation are serious threats to road traffic safety.

Traffic flow and road network density are important characteristics of road transportation. Shandong Province is located in the core area of land transportation in China and has a well-developed road network. As of 2022, the total road mileage in Shandong Province reached 291,800 kilometers (km), ranking fourth in China. Simultaneously, Shandong Province is an economically developed province, with many motor vehicles and high traffic flow on highways and arterial roads. Besides, the total population of Shandong Province has reached 101.78 million people, which represents a high population density. Studies have demonstrated that the higher the population density, the more likely the occurrence of traffic injuries, especially serious RTIs (16). Considering the aforementioned factors, the risk of RTIs and fatalities in Shandong Province is exceedingly high.

While spatio-temporal patterns of road traffic injuries (RTIs) have been studied in other Chinese contexts (17, 18), recent evidence underscores the need for updated region-specific analyses. A study on the impact of transportation investments reveals significant regional disparities in road traffic fatalities across China (19). Meanwhile, research on non-motorized vehicles confirms the increasing role of e-bikes in traffic injuries within dense urban environments (20). Given the considerable socioeconomic heterogeneity across Chinese regions, a comprehensive analysis focusing on Shandong Province—an area with distinct economic and geographical characteristics—remains lacking. This is particularly true for studies covering the most recent decade that differentiate risks among all road user types.

Therefore, this study aims to analyze the temporal and spatial distribution and spatio-temporal clustering of road traffic fatalities in Shandong Province from 2012 to 2022. Specifically, it identifies distinct, evolving spatio-temporal risk patterns for specific road user groups—highlighting the rising threat to non-motorized drivers and the unique high-risk environment for motorized drivers in the Jiaodong Peninsula. This analysis is crucial for developing customized prevention strategies, with significant practical implications for reducing fatalities.

## 2 Materials and methods

### 2.1 Data sources

The timeframe of this study was from January 1, 2012 to December 31, 2022. Data on road traffic fatalities in Shandong Province were obtained from the Chinese Center for Disease Control and Prevention Population Death Information Registration Management System (21). The data collected included age, gender, education, marital status, and International Classification of Diseases (ICD)-10 codes. The study participants were categorized according to ICD-10 codes into four subgroups: pedestrians, non-motorized drivers, motorized drivers, and passengers. The personal information of all participants was de-identified to ensure confidentiality and protect their privacy.

### 2.2 Quality control

The death registration system in Shandong Province operates under strict quality control protocols jointly formulated by the Departments of Health and Public Security. These protocols ensure high standards for data completeness and accuracy.

To specifically address and quantify under-reporting, a known limitation in passive surveillance systems, the Shandong Provincial CDC conducts routine under-reporting surveys following the national protocol established by the China CDC. A 2018 study by the China CDC indicated that the initial completeness of death registration in Shandong Province was 83.1% (22). To correct for this, under-reporting surveys are conducted every 3 years using the capture-recapture method across the province’s 31 national disease surveillance points. The mortality data are then adjusted based on the survey results. After this correction, the data achieve the quality standards required for Chinese cause-of-death data, with an accuracy rate of ≥95%. Therefore, the final dataset used for our analysis consists of these adjusted figures, which provides a more robust and reliable estimate of the true burden of road traffic fatalities in Shandong Province compared to raw, unadjusted counts.

### 2.3 Statistical analysis

A database on road traffic fatalities was established following data cleansing. Composition ratios were used to describe the basic situation of the road traffic fatality population in Shandong Province from 2012 to 2022, and the chi-square test was used for the analysis of variance. Excel 2016 software was employed to draw a line graph of the temporal distribution of the number of deaths in each group. The global and local spatial autocorrelation of the number of fatalities in each group was analyzed using Geoda software to analyze clusters or outliers, as well as areas where spatial hotspots were located. Spatio-temporal aggregation was analyzed using the Poisson model with Monte Carlo hypothesis testing within SaTScan v10.1.2 software. The time aggregation unit was set to ‘year’. To thoroughly explore all potential clusters without pre-defined constraints, the maximum spatial cluster size and the maximum temporal cluster size were both set to include 100% of the population at risk. The test level was set to *α* = 0.05.

## 3 Results

### 3.1 Basic situation of road traffic fatalities in Shandong Province

The total number of deaths due to road traffic accidents in Shandong Province from 2012 to 2022 was 176,129, with an average age of 52.65 ± 17.94 years old. Over 40 years old was the main age group, accounting for 77.35% (136,239/176,129), and the gender ratio of men to women was about 2.77:1 (129,405/46,724). In terms of types of deaths, pedestrians were the main population, accounting for 55.18% of the total population (97,188/176,129), followed by motorized drivers (21.42%, 37,724/176,129), non-motorized drivers (14.74%, 25,968/176,129), and passengers (8.66%, 15,249/176,129). In terms of age, 60 years and above was the main age group for pedestrians and non-motorized drivers, whereas passengers and motorized drivers were in the 40–59 age group. In terms of gender, males were higher than females in the subgroup, and they were also more prevalent among motorized drivers. In terms of education and marital status, all subgroups revealed that individuals with a junior high school and below and those who were married were the main groups (Table 1).

**Table 1:** Basic demographics of different groups of road traffic fatalities (n, %).

Variables	*N*	Pedestrians	Non-motorized drivers	Passengers	Motorized drivers
Age(years)					
<20	7,093	4,841 (4.98)	684 (2.63)	1,170 (7.67)	398 (1.06)
20–39	32,797	15,986 (16.45)	3,096 (11.92)	3,667 (24.05)	10,048 (26.64)
40–59	67,727	35,053 (36.07)	10,010 (38.55)	5,853 (38.38)	16,811 (44.56)
≥60	68,512	41,308 (42.50)	12,178 (46.90)	4,559 (29.90)	10,467 (27.74)
Gender					
Male	129,405	69,014 (71.01)	17,803 (68.56)	10,505 (68.89)	32,083 (85.05)
Female	46,724	28,174 (28.99)	8,165 (31.44)	4,744 (31.11)	5,641 (14.95)
Marital status					
Unmarried	17,201	9,856 (10.14)	1961 (7.55)	2019 (13.24)	3,365 (8.92)
Married	146,323	79,630 (81.93)	22,264 (85.74)	12,127 (79.53)	32,302 (85.63)
Divorced or widowed	10,065	5,894 (6.06)	1,577 (6.07)	784 (5.14)	1810 (4.80)
Others	2,540	1808 (1.87)	166 (0.64)	319 (2.09)	247 (0.65)
Education					
Junior high school and below	140,199	78,183 (80.45)	21,471 (82.68)	11,263 (73.86)	29,282 (77.62)
High school	29,865	15,320 (15.76)	3,826 (14.73)	3,130 (20.53)	7,589 (20.12)
College and above	1,512	788 (0.81)	200 (0.77)	188 (1.23)	336 (0.89)
Unclear	4,553	2,897 (2.98)	471 (1.82)	668 (4.38)	517 (1.37)
Total	176,927	97,188	25,968	15,249	37,724

### 3.2 Temporal distribution

**Figure 1:**
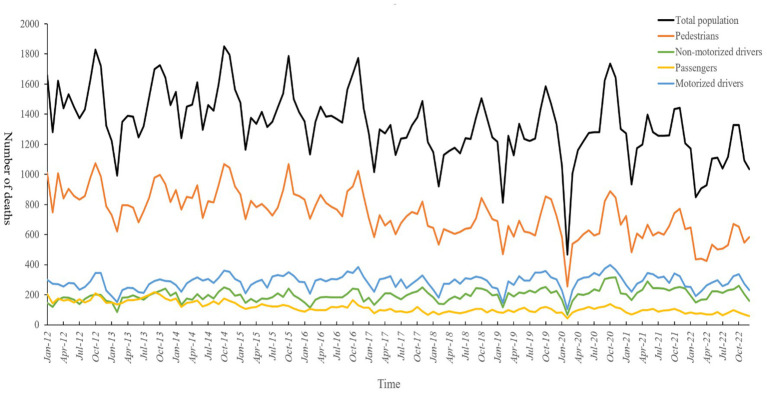
Temporal distribution of road traffic fatalities in Shandong Province, 2012–2022.

As displayed in Figure 1, there were cyclical trends in the total population, pedestrians, non-motorized drivers, and passengers, with autumn being the season with the highest numbers. The total population, pedestrians, and passengers showed a decreasing trend over the years, while non-motorized drivers gradually increased, and motorized drivers exhibited no significant trend. In February 2020, all groups experienced a dramatic decrease due to the drastic impact of the COVID-19 epidemic prevention and control requirements on transportation activities. Further analysis revealed statistically significant differences in the temporal distributions of the groups in terms of leave in lieu (*χ^2^* = 12.247, *p* = 0.007) and vacation (*χ^2^* = 17.599, *p* = 0.001), where the share of passengers was higher than that of the other groups (see Table 2).

**Table 2:** Comparison of differences in time of death between groups of road traffic fatalities.

Variables	*N*	Pedestrians	Non-motorized drivers	Passengers	Motorized drivers	*χ^2^*	*p*-value
Working days						6.460	0.091
Yes	117,871	64,940 (66.82)	17,523 (67.48)	10,120 (66.37)	25,288 (67.03)		
No	58,258	32,248 (33.18)	8,445 (32.52)	5,129 (33.63)	12,436 (32.97)		
Leave in lieu						12.247	**0.007**
Yes	3,102	1718 (1.77)	456 (1.76)	315 (2.07)	613 (1.62)		
No	173,027	95,470 (98.23)	25,512 (98.24)	14,934 (97.93)	37,111 (98.38)		
Vacation						17.599	**0.001**
Yes	13,817	7,620 (7.84)	1928 (7.42)	1,300 (8.53)	2,887 (7.65)		
No	163,110	89,568 (92.16)	24,040 (92.58)	13,949 (91.47)	34,837 (92.35)		
Total	176,927	97,188	25,968	15,249	37,724		

Bold values indicates *p* < 0.05, the difference is statistically significant.

### 3.3 Spatial distribution

The spatial distribution of road traffic fatalities in Shandong Province from 2012 to 2022 indicated that Linyi (23,959) was the city with the highest number of deaths in the total population, and Dongying (4,030) was the lowest, and the number of deaths in Linyi was about six times higher than that in Dongying. Besides, Linyi (12,165) was the city with the highest number of pedestrians, and Dongying (1,550) was the lowest. Furthermore, Linyi (4,095) was the city with the highest number of non-motorized drivers, and Dongying (626) was the lowest. Lastly, Linyi (2,062) had the highest number of passengers, while Weihai (387) had the lowest. Weifang (5,880) had the highest number of motorized driver deaths, while Liaocheng (881) had the lowest (see Table 3).

**Table 3:** Regional distribution of road traffic fatalities in Shandong Province, 2012–2022 (*n*, %).

City	*N*	Pedestrians	Non-motorized drivers	Passengers	Motorized drivers
Jinan	11,100	5,496 (5.66)	1,696 (6.53)	1,070 (7.02)	2,838 (7.52)
Qingdao	8,656	5,644 (5.81)	984 (3.79)	430 (2.82)	1,598 (4.24)
Zibo	6,283	2,940 (3.03)	1,165 (4.49)	722 (4.73)	1,456 (3.86)
Zaozhuang	8,876	5,416 (5.57)	1,270 (4.89)	905 (5.93)	1,285 (3.41)
Dongying	4,030	1,550 (1.59)	626 (2.41)	458 (3.00)	1,396 (3.70)
Yantai	12,261	5,412 (5.57)	1,469 (5.66)	1,096 (7.19)	4,284 (11.36)
Weifang	17,316	6,749 (6.94)	2,695 (10.38)	1992 (13.06)	5,880 (15.59)
Jining	16,069	11,369 (11.70)	2040 (7.86)	1,168 (7.66)	1,492 (3.96)
Tai’an	10,113	3,984 (4.10)	1938 (7.46)	845 (5.54)	3,346 (8.87)
Weihai	5,762	2,869 (2.95)	896 (3.45)	387 (2.54)	1,610 (4.27)
Rizhao	6,115	3,175 (3.27)	1,193 (4.59)	544 (3.57)	1,203 (3.19)
Linyi	23,959	12,165 (12.52)	4,095 (15.77)	2062 (13.52)	5,637 (14.94)
Dezhou	9,917	7,175 (7.38)	1,149 (4.42)	612 (4.01)	981 (2.60)
Liaocheng	12,342	9,261 (9.53)	1,292 (4.98)	908 (5.95)	881 (2.34)
Binzhou	8,287	3,966 (4.08)	1,618 (6.23)	1,020 (6.69)	1,683 (4.46)
Heze	15,043	10,017 (10.30)	1842 (7.09)	1,030 (6.77)	2,154 (5.69)

### 3.4 Spatial autocorrelation analysis

Global spatial autocorrelation analyses of road traffic deaths in Shandong Province for each group from 2012 to 2022 demonstrated that the global spatial autocorrelation of passengers (Moran’s *I* = −0.509, *p* = 0.038) was statistically significant. However, the total population (Moran’s *I* = −0.323, *p* = 0.321), pedestrians (Moran’s *I* = −0.008, *p* = 0.796), non-motorized drivers (Moran’s *I* = −0.286, *p* = 0.248), and motorized drivers (Moran’s *I* = −0.273, *p* = 0.352) showed no statistical significance. A negative but non-significant Moran’s I value suggests a tendency towards a dispersed spatial pattern, but this pattern is not strong enough to be statistically distinguished from a random distribution.

The local spatial autocorrelation analysis was conducted to investigate the potential local spatial clustering. The local spatial autocorrelation analysis revealed statistically significant spatial clustering patterns. Specifically, for the total population, Rizhao and Zaozhuang formed a significant ‘Low-High’ cluster (cold spot surrounded by hotspots), while Binzhou constituted a significant ‘Low-Low’ cluster (cold spot). This indicates that the high fatalities in Rizhao and Zaozhuang are surrounded by areas with similarly high numbers, forming a regional hotspot, whereas Binzhou is part of a larger low-risk region. Rizhao and Zaozhuang were identified as low-high cluster areas for pedestrians. Rizhao exhibited a low-high cluster area among non-motorized drivers, whereas Binzhou showed a high-low cluster area for this group. Rizhao also demonstrated a low-high cluster area for passengers, in contrast to Weifang, which presented a high-low cluster area for passengers. Yantai was identified as a low-low cluster area for passengers, whereas Zaozhuang exhibited a high-high cluster area for this group. Furthermore, Rizhao and Qingdao were classified as low-high cluster areas for motorized drivers.

### 3.5 Hotspot analysis of space

The results of the hotspot analysis of all groups of road traffic fatalities in Shandong Province from 2012 to 2022 indicated that Zaozhuang and Rizhao were hotspots for the total population, Zaozhuang and Heze were hotspots for pedestrians, Rizhao was a hotspot for non-motorized drivers, Zaohuang was a hotspot for passengers, Yantai was a cold spot for passengers, and Qingdao and Rizhao were hotspots for motorized drivers.

### 3.6 Spatio-temporal cluster analysis

The results of the spatio-temporal clustering analysis showed that two clusters were detected in the number of deaths in the total population. The first and second aggregation areas were both from January 2012 to June 2017. The radius of the first aggregation area was 192.4 km, and the cluster area included Linyi, Zaozhuang, Rizhao, Jining, Tai’an, Zibo, and Weifang. The regional radius of the second cluster area was 318.44 km, and the cluster locations included Dongying, Dezhou, and Binzhou. There were three clusters of the number of deaths of pedestrians: the first and third clusters overlapped the total population in terms of location, timeframe, and radius, and the second cluster of pedestrians was LiaoCheng, with a radius of 0 km and a timeframe from January 2013 to June 2018. The number of deaths of non-motorized drivers had two clusters: the first cluster occurred from July 2017 to December 2020 and included the locations of Linyi, Zaozhuang, Rizhao, Jining, Tai’an, Zibo, and Weifang; the second cluster occurred from July 2016 to December 2021 and included the location of Dongying, Dezhou, and Binzho. The spatio-temporal cluster of the number of passengers was perfectly aligned with the location, timeframe, and radius of the total population. There were two clusters of motorized drivers. The first cluster occurred from July 2013 to December 2018, with locations including Qingdao, Rizhao, Weifang, Yantai, Linyi, Weihai, Zibo, and Tai’an, with a radius of 296.15 km. The second cluster occurred from July 2020 to July 2021, with a location in Dezhou (see Table 4).

**Table 4:** Results of spatio-temporal aggregation analysis of road traffic fatalities in Shandong Province, 2012–2022.

Classifications	Cluster	Geographical location	Timeframe	Radius (kilometers)	*RR*	*LLR*	*p*-value
Total population	1	Linyi, Zaozhuang, Rizhao, Jining, Tai’an, Zibo, Weifang	2012/01–2017/06	192.4	3.40	21,168	<0.001
	2	Dongying, Dezhou, Binzhou	2012/01–2017/06	318.44	2.88	4,885	<0.001
Pedestrians	1	Linyi, Zaozhuang, Rizhao, Jining, Tai’an, Zibo, Weifang	2012/01–2017/06	192.40	3.28	10,825	<0.001
	2	Liaocheng	2013/01–2018/06	0.00	4.48	3,898	<0.001
	3	Dongying, Dezhou, Binzhou	2012/01–2017/06	318.44	3.22	3,524	<0.001
Non-motorized drivers	1	Linyi, Zaozhuang, Rizhao, Jining, Tai’an, Zibo, Weifang	2017/07–2020/12	192.40	3.54	3,482	<0.001
	2	Dongying, Dezhou, Binzhou	2016/07–2021/12	318.44	2.81	688	<0.001
Passengers	1	Linyi, Zaozhuang, Rizhao, Jining, Tai’an, Zibo, Weifang	2012/01–2017/06	192.40	4.37	2,907	<0.001
	2	Dongying, Dezhou, Binzhou	2012/01–2017/06	318.44	3.40	624	<0.001
Motorized drivers	1	Qingdao, Rizhao, Weifang, Yantai, Linyi, Weihai, Zibo, Tai’an	2013/07–2018/12	296.15	4.00	7,066	<0.001
	2	Dezhou	2020/07–2021/07	0.00	1.61	15	<0.001

## 4 Discussion

Since the invention of automobiles in the late 19th century, over 50 million people have died from road traffic crashes worldwide (23), and RTIs have become a serious public health problem. Shandong Province is a typical region for road traffic crashes in China (24), which have a significant impact on people’s health and property safety. We analyzed the temporal and spatial distribution and spatio-temporal aggregation of road traffic fatalities in Shandong Province between 2012 and 2022 using the data on road traffic fatalities in the Population Death Information Registration and Management System of the Chinese Center for Disease Control and Prevention. The results can provide a scientific basis for developing intervention strategies and preventive and control measures for road traffic crashes in Shandong Province.

This study found that pedestrians were the main group involved in road traffic fatalities, similar to previous research findings (25, 26) but different from the results of Curtis et al.’s (27) study in Australia. The death of pedestrians is not only linked to their inherent vulnerability to traffic activities but also to population density, socioeconomic status, and modes of transportation. In Shandong Province, the number of male road traffic fatalities far exceeds that of females, potentially attributed to the social and occupational dynamics within traffic activities. Males are the most active and productive group in road traffic activities in China, resulting in a significantly higher number of male traffic personnel compared to females (28). This also makes them the main victims of road traffic crashes. Furthermore, even if males and females play the same roles in traffic activities, females tend to be more cautious and less prone to errors in road traffic activities (29). This finding is closely associated with the level and manner of female participation in traffic activities.

The proportion of people aged 40 years and older was the highest among all groups. This may be linked to the age structure and labor participation rate in China, as well as to physiological functions. People in this age group are susceptible to serious injuries and even death from road traffic crashes due to limitations in their physical functioning, such as reduced mobility and poor reflexes (30). The results of this study showed that non-motorized drivers were older than other subgroups, similar to the findings of Li et al. (31). Due to the lightweight and easy accessibility of non-motorized vehicles, they are used by a wider range of people, especially older age groups. This study also found that people with junior high school and below were the main group of road traffic fatalities. Education is an important factor that influences road traffic crashes (32), as lack of education may limit people’s understanding of traffic signs and regulations, leading to risky behaviors.

This study found that autumn was the highest season for all road traffic fatality groups, similar to the results of some studies (33, 34). Autumn marks the transition from hot to cold temperatures. Previous studies have shown that temperature is a negatively correlated factor with road traffic fatalities in Shandong Province (35). During the initial phase of autumn, the lingering heat may induce irritability and distraction among drivers, potentially resulting in hazardous driving conditions (36). Furthermore, as temperatures drop gradually, the increase in outdoor activities increases the risk of traffic safety (37). Additionally, autumn heralds the widespread harvesting of crops in Shandong Province, resulting in a substantial surge in using agricultural vehicles, which exacerbates challenges to traffic safety, The research of Chen et al. (38) also shows that the frequency of traffic accidents of agricultural vehicles in autumn is high, accounting for 43.4%. In terms of vacation and leave in lieu, the percentage of passengers was higher than in other groups. During vacations, intercity mobility increases, where family-oriented self-driving and public transportation are the predominant modes of travel (39), leading to a larger proportion of individuals being passengers compared to typical circumstances. Leave in lieu disrupts the regular routine, which can have a negative impact on biorhythms, potentially resulting in fatigue, insomnia, or reduced mental performance. Changes in routine often contribute to sleep disorders, which, in turn, increase the likelihood of car accidents by a factor of 2.96 compared to adhering to a normal routine (40). Establishing consistent patterns of rest and relaxation is an effective measure for preventing road traffic collisions.

According to the results of a study by the China CDC, the road traffic mortality rate in China decreased by 21.8% in 2017 compared to 1990 (41). Our study revealed similar findings, with the number of road traffic fatalities in Shandong Province showing a decreasing trend from 2012 to 2022. Various factors can influence the occurrence of road traffic crashes, with laws and regulations playing a crucial role in determining the outcomes. In 2011, China formally incorporated drunk driving as a criminal offense under the charge of dangerous driving into its Penal Code. Previous study have shown that the implementation of this policy can reduce the annual mortality rate of road traffic in Shandong Province by 6.48% (42). According to China’s Ministry of Public Security (2011–2021), the country witnessed a significant increase of 181 million motor vehicles and 259 million drivers. Pedestrians and passengers, compared to motorized drivers, typically have inadequate protection and diminished awareness of road hazards when engaged in traffic situations. Consequently, they are more susceptible to injuries and fatalities owing to severe collisions. Implementing stringent penalties through laws and regulations is effective in deterring dangerous driving behaviors, thus promoting a safer road environment for all, particularly vulnerable road users such as pedestrians and passengers. Moreover, enhanced education plays a pivotal role in deepening drivers’ comprehension of traffic regulations and signs, thereby mitigating the occurrence of road accidents (43). Furthermore, an inevitable increase in the volume of motor vehicles due to economic advancement has been observed. Research indicates a 2.83% increase in traffic accidents for every 1% increase in motor vehicle ownership (44). Enhancing the equipment and structural specifications of vehicle safety devices is crucial for mitigating injuries sustained by drivers and passengers in vehicle collisions. Establishing a scientific and practical road maintenance management system not only facilitates the creation of a conducive road environment but also minimizes the frequency of road traffic crashes (45).

Despite the general decrease in road traffic fatalities in Shandong Province, the number of victims of non-motorized vehicles, particularly e-bikes, has been increasing. E-bikes are popular due to their lightweight and speed. Because e-bikes exhibit the characteristics of both motor vehicles and bicycles, the nature of road traffic accidents involving them is unique (46). They can behave like bicycles in collisions with motor vehicles but are more akin to motor vehicles in accidents involving bicycles or pedestrians. By the end of 2022, China became the global leader in the social ownership of e-bicycles, boasting an estimated total of approximately 350 million units. As a result, the rise in traffic violations associated with e-bicycles, including infractions such as traveling against the flow of traffic and disregarding traffic signals, has emerged as a pervasive and multifaceted challenge that demands attention from governmental authorities across various levels. The decline in using public transportation due to the pandemic control measures enacted during the COVID-19 outbreak has contributed to a notable increase in road traffic crashes involving e-bicycles, amplifying existing traffic safety hazards (47, 48). Besides, the elevated frequency of such crashes compounded the overall risk factors correlated with traffic safety. Implementing road safety education, especially for non-motorized drivers, plays a pivotal role in reducing the number of road traffic fatalities within the community.

The results of the global spatial autocorrelation analysis disclosed that in Shandong Province from 2012 to 2022, only passengers’ road traffic fatalities were globally spatially correlated, and no global spatial correlation was found for the total population, pedestrians, non-motorized drivers, and motorized drivers. Local spatial autocorrelation analysis revealed non-random spatial clustering of road traffic fatalities in all groups in Shandong Province. This study was the first to analyze the spatio-temporal aggregation of road traffic fatalities in Shandong Province, showing that all groups had different spatio-temporal aggregations, except for the total population, pedestrians, and passengers, which were aggregated in the same geographic location and timeframe, from 01/2012 to 06/2017. With economic development, the spatio-temporal aggregation of the total population, pedestrians, and passengers can be altered by increased vehicle safety (49), improved road traffic conditions (50), and changes in inter-regional population densities due to accelerated population mobility (51, 52).

Our findings reveal that the Jiaodong Peninsula (including Qingdao, Yantai, Weihai, etc.) constitutes a distinct and high-risk spatio-temporal cluster for motorized drivers, a pattern that can be deconstructed through its unique regional characteristics. This region hosts numerous major seaports in China and serves as a pivotal distribution hub for maritime cargo transportation. Heavy freight vehicles can destabilize traffic flow, leading to an increased risk of road traffic crashes for small vehicles (53). Second, the Jiaodong Peninsula is influenced by the maritime climate, and unfavorable weather affecting road traffic safety, such as high winds (54) and precipitation (55), is more prevalent than in inland areas. Lastly, topography plays a crucial role in influencing road traffic fatalities among drivers (28, 56). The Jiaodong Peninsula primarily features plains and hills, and roads built to accommodate the topography can induce driver visual fatigue, potentially resulting in unsafe driving behaviors. The confluence of these economic, environmental, and topographical factors creates a synergistic high-risk environment, establishing the Jiaodong Peninsula as a critical priority for targeted motor vehicle safety interventions.

Our findings advocate for a transition from uniform provincial policies to precision interventions targeting specific high-risk zones and road user groups. In southwestern Shandong, where non-motorized fatalities are emerging as a critical concern, immediate measures should include mandatory helmet laws for e-bike riders and the development of protected bicycle lanes. Concurrently, the Jiaodong Peninsula requires a focus on its motorized driver cluster through dynamic enforcement in freight corridors, driver advisories for adverse weather conditions, and road design improvements to mitigate topography-related risks. These targeted strategies should be complemented by enhanced pedestrian infrastructure in identified urban hotspots like Zaozhuang and Rizhao. Implementing this spatially-explicit approach will enable more efficient resource allocation and significantly advance road safety outcomes across the province.

## 5 Limitations

This study thoroughly investigated the spatial and temporal distributions and aggregation characteristics of road traffic fatalities in Shandong Province, China. However, certain limitations of this study must be acknowledged. Firstly, the accuracy of the ICD-10 codes needs to be at least 95% for proper management of population death information registration; consequently, there is a possibility of incorrect ICD-10 coding. Secondly, although the data have been corrected based on periodic under-reporting surveys, a possibility of residual under-reporting or misclassification cannot be entirely ruled out, as is common in all passive surveillance systems. Finally, the spatial resolution of our analysis was confined to the city level, which precluded the identification of risk factors at more granular scales, such as specific road segments or intersections. Additionally, the absence of detailed crash circumstance data (e.g., specific vehicle types involved, lighting conditions) in our dataset limited our ability to investigate the precise mechanisms behind the identified spatio-temporal clusters. Despite these constraints, this study offers valuable scientific insights into identifying high-incidence areas and periods for road traffic fatalities in Shandong Province, contributing a theoretical foundation for formulating preventive and control strategies for road traffic crashes.

## 6 Conclusion

This study provides a decade-long spatio-temporal analysis that uncovers two critical and novel insights for road safety policy in Shandong: the emerging epidemic of non-motorized driver fatalities in the southwest and the persistent, multifactorial hotspot of motorized driver fatalities in the Jiaodong Peninsula. Instead of uniform provincial-wide measures, our findings advocate for a precision prevention framework. Resources should be prioritized for high-risk areas and periods identified in this study. This study not only showed the epidemiological characteristics of road traffic fatalities in Shandong but also provides an evidence-based foundation for developing targeted, cost-effective interventions. Based on this study, potential directions for future research include: integrating geocoded crash data to enable street-level risk analysis; employing mixed-methods approaches to better understand the determinants behind persistent clusters; and conducting intervention studies to evaluate the effectiveness of targeted measures in high-risk zones. The methodology and findings offer valuable insights for road safety planning in similar regions.

## Data Availability

The raw data supporting the conclusions of this article will be made available by the authors, without undue reservation.
